# Assessment of genotyping markers in the molecular characterization of a population of clinical isolates of *Fusarium* in Colombia

**DOI:** 10.7705/biomedica.5869

**Published:** 2022-03-01

**Authors:** Valeria Velásquez-Zapata, Katherine Palacio-Rúa, Luz E. Cano, Adelaida Gaviria-Rivera

**Affiliations:** 1 Program in Bioinformatics and Computational Biology, Iowa State University, Ames, IA, USA Iowa State University Iowa State University Ames, IA USA; 2 Department of Plant Pathology and Microbiology, Iowa State University, Ames, IA, USA Iowa State University Iowa State University Ames, IA USA; 3 Laboratorio Integrado de Medicina Especializada, Facultad de Medicina, IPS Universitaria, Universidad de Antioquia, Medellin, Colombia Universidad de Antioquia Universidad de Antioquia Medellin Colombia; 4 Grupo de Micología Médica y Experimental, Corporación para Investigaciones Biológicas, Medellin, Colombia Corporación para Investiga. Biológicas Corporación para Investigaciones Biológicas Medellin Colombia; 5 Escuela de Microbiología, Universidad de Antioquia, Medellin, Colombia Universidad de Antioquia Universidad de Antioquia Medellin Colombia; 6 Escuela de Biociencias, Facultad de Ciencias, Universidad Nacional de Colombia, Medellin, Colombia Universidad Nacional de Colombia Universidad Nacional de Colombia Medellin Colombia

**Keywords:** Fusarium, fusariosis, genotyping techniques, bacteriophage M13, elongin, genetics, population, DNA fingerprinting, Fusarium, fusariosis, técnicas de genotipificación, bacteriófago M13, elonguina, genética de población, dermatoglifia del ADN

## Abstract

**Introduction:**

*Fusarium* is a very heterogeneous group of fungi, difficult to classify, with a wide range of living styles, acting as saprophytes, parasites of plants, or pathogens for humans and animals. Prevalence of clinical fusariosis and lack of effective treatments have increased the interest in the precise diagnosis, which implies a molecular characterization of *Fusarium* populations.

**Objective:**

We compared different genotyping markers in their assessment of the genetic variability and molecular identification of clinical isolates of *Fusarium*.

**Materials and methods:**

We evaluated the performance of the fingerprinting produced by two random primers: M13, which amplifies a minisatellite sequence, and (GACA)_4_, which corresponds to a simple repetitive DNA sequence. Using the Flunter Gaston Discriminatory Index (HGDI), an analysis of molecular variance (AMOVA), and a Mantel test, the resolution of these markers was compared to the reference sequencing-based and PCR genotyping methods.

**Results:**

The highest HGDI value was associated with the M13 marker followed by (GACA)_4_. AMOVA and the Mantel tests supported a strong correlation between the M13 classification and the reference method given by the partial sequencing of the transcription elongation factor 1-alpha (TEF1-α) and rDNA 28S.

**Conclusion:**

The strong correlation between the M13 classification and the sequencing- based reference together with its higher resolution demonstrates its adequacy for the characterization of *Fusarium* populations.

## Resumen

**Introducción.:**

*Fusarium* es un grupo heterogéneo de hongos, difícil de clasificar y con una amplia gama de estilos de vida, que actúa como saprofito, parásito de plantas o patógeno de humanos y animales. La prevalencia de la fusariosis clínica y la falta de tratamientos han incrementado el interés en su diagnóstico preciso, lo que conlleva la caracterización molecular de las poblaciones.

**Objetivo.:**

Comparar marcadores de genotipificación en la evaluación de la variabilidad genética e identificación de aislamientos clínicos de *Fusarium*.

**Materiales y métodos.:**

Se evaluó la huella genética producida por dos cebadores aleatorios: M13, que amplifica una secuencia minisatélite, y (GACA)_4_, que corresponde a una secuencia repetitiva de ADN. Utilizando el índice discriminatorio de Hunter Gaston (HGDI), el análisis de varianza molecular (AMOVA) y una prueba de Mantel, se comparó la resolución de estos marcadores con métodos de genotipificación basados en secuenciación y PCR.

**Resultados.:**

El mayor HGDI se asoció con el marcador M13, seguido de (GACA)_4_. Las pruebas AMOVA y Mantel mostraron correlación entre las clasificaciones obtenidas con M13 y la referencia basada en la secuenciación parcial del factor de elongación de transcripción 1-alfa (TEF1-α) y el ADNr 28S.

**Conclusión.:**

La fuerte correlación entre la clasificación obtenida con M13 y el método de referencia, así como su alta resolución, demuestran su idoneidad para la caracterización de poblaciones de *Fusarium*.

*Fusarium* (Ascomycota, Pezizomycotina, Sordariomycetes, Hypocreomycetidae, Hypocreales, Nectriaceae) is a ubiquitous fungus widely distributed in soil, plants, and organic substrates. Currently, infections by *Fusarium* are the second most frequent cause by environmental molds and they are associated with high rates of mortality in immunosuppressed patients including those with AIDS and cancer, prolonged use of corticosteroids, and transplanted organs ^1^^-^^2^. The pathogenicity by *Fusarium* is associated with the production of mycotoxins, proteases, collagenases, and keratinases ^3^^-^^4^.

Pathologies associated with *Fusarium* in humans include *Fusarium* keratitis, onychomycosis, endophthalmitis, peritonitis, invasive *Fusarium* disease, and skin infections. Less frequent infections include osteomyelitis, arthritis, otitis, sinusitis, and brain abscesses ^1^^-^^2^^,^^5^. The most common pathology among immunosuppressed patients is invasive or disseminated reaching a 70% to 100% mortality and infection sources are skin and nail lesions ^6^. This invasive nature, as well as its role in fungal keratitis and its difficult treatment, increase its prevalence in the clinical setting ^7^^-^^8^. The most frequent human pathogens of *Fusarium* are the species complexes: F. *solani* (Mart.) Sacc. (FSSC), considered as the most resistant, followed by F. oxysporum Smith & Swingle (FOSC) ^2^^,^^9^. Other species such as *F. dimerum* Penz. 1882 (FDSC) ^10^, *F. proliferatum* (Matsush.) Nirenberg 1976 ^11^, *F. incarnatum-equiseti* (Desm.) Sacc. 1886 (FIESC) ^12^,*F.sacchari* (E. J. Butler & Hafiz Khan) W. Gams 1971 ^13^, *F.fujikuroi* Nirenberg 1976 (FFSC) ^12^, *F. chlamydosporum* Wollenw & Reinking 1925 (FCSC) ^12^, *F. moniliforme* J. Sheld 1904 ^14^^,^^15^ and *F*. sporotrichioides Sherb. 1915 ^15^ have also been reported.

The taxonomy of the species complexes in this genus is not a trivial task and there have been many proposals of reclassifying it into multiple genera ^16^. In the last years, FSSC has been the object of a debate on its reclassification to the genus *Neocosmospora*^17^ with arguments in favor of each option. However, the large amount of information about fusariosis shows the importance of keeping the FSSC classification for medical professionals ^16^. In Colombia, the identification of *Fusarium* at the species level for epidemiological purposes is not common ^18^^,^^19^, although, in the last years, there have been more studies to characterize clinical isolates at this level ^20^^-^^23^. The lack of this type of characterization has been a serious problem for physicians as proper identification is necessary for an adequate treatment ^24^. These fungi have variable susceptibility to antifungals and some of them are resistant to all the available treatments ^9^^,^^12^^,^^25^. Furthermore, it is necessary to compare the susceptibility of each isolate with a given genotype given the high variability at the intraspecific level ^8^. This implies characterizing the populations at the highest resolution possible while keeping the simplicity and efficiency required for diagnosis in clinical laboratories in the country.

*Fusarium* identification traditionally relied on morphological characters; however, these vary at the intraspecific level and are influenced by culture conditions ^26^. On the other hand, the description of some characters is subjective and the procedure can be lengthy and expensive and it needs trained mycologists ^14^^,^^26^, which prompted the development of molecular techniques for the identification of the genus ^26^. DNA-based techniques are useful for determining *Fusarium* taxonomy as they do not have the difficulties arising from the use of morphological characters and, besides, they reveal the genetic diversity ^26^. Different molecular markers have been proposed for *Fusarium* genotyping and classification including random species-specific primers and the sequencing of different genes ^5^^,^^27^^-^^30^. Currently, the highest resolution marker is the translation elongation factor 1-α (TEF1-α) gene ^31^ now complemented with multilocus sequence typing (MLST) through the addition of two partial sequences from the two largest DNA-directed RNA polymerase subunits (RPB1 and RPB2) ^29^^,^^32^. Despite these resources, there is still a need for other molecular methods to complement current approaches and provide more information about the genetic diversity of the isolates and their correlation with phenotypical traits ^23^. Therefore, it is necessary to evaluate molecular markers for the identification of *Fusarium* species supplying enough information for phylogenetic inference, population genetics, and epidemiology ^8^.

Our study centered on genotyping clinical isolates of this group of fungi obtained from patients in Medellin, Colombia, to establish the utility of two molecular markers which may provide resolution at the species level and measure the genetic diversity of *Fusarium* populations. We also evaluated the correlation between the genotypes and relevant phenotypic traits for epidemiological purposes including the isolation tissue and the susceptibility to antifungals. We evaluated two primers according to their gel fingerprints: a mini-satellite probe generated from the M13 bacteriophage and the (GACA)_4_ simple DNA repeat sequence ^5^. We compared their performance using sequencing and PCR-based methods already reported by our group ^22^^,^^23^. The genotyping results using these markers were clustered and the classifications compared with other molecular markers including the partial sequencing of the 28S ribosomal subunit (rDNA 28S) ^30^ and the transcription elongation factor 1-alpha (TEF1-α), widely accepted as reference for this group, as well as the PCR-based identification using 0X31/0X32 primers ^27^ and Fusofor/Fusorev ^28^.

## Materials and methods

### Isolates

We obtained isolates from 101 patients referred by health providers to the *Laboratorio de Micología Médica y Experimental* at the *Corporación* para *Investigaciones Biológicas* (CIB) in Medellin after obtaining their informed consent. Before we took the samples, we gathered the data on their sex, age, place of residence, and previous use of antifungals. The samples were then obtained from hands and feet fingernails, cornea, sinuses, skin, and secretions by non-invasive procedures warranting the chain of custody at the institutions to avoid cross contamination. Samples were cultured in three different media: Sabouraud agar (Thermo Scientific, USA), potato dextrose agar (Thermo Scientific, USA), and mycosel agar (Fisher Scientific, USA) and 10 inoculation points, at room temperature for one to three weeks. *Fusarium* was considered the etiological agent based on two criteria: ^1^ the growth of the isolate in the media was observed in more than five of the inoculation points and ^2^ the same isolate was obtained in at least two of the three media. Once growth was observed, the macroscopic colonies identified as *Fusarium* sp. were stored in sterile vials ^25^.

### DNA extraction and purification

The isolates were cultured for 10 days in Sabouraud medium (Thermo Scientific, USA) and, subsequently, a portion of the mycelia was macerated with liquid nitrogen in a laminar flow chamber for DNA extraction with the DNeasy Plant Mini-kit (Qiagen) with the following modifications: a vortex of the sample with buffer API for 30 minutes and incubation with RNAse A for another 30 minutes. The DNA was suspended in 80 µl of water for subsequent quantification at 260 nm in a Nanodrop 1000 spectrophotometer (Thermo Scientific, USA).

### Molecular genotyping

The molecular genotyping of *Fusarium* isolates was carried out by PCR using two random primers: a 15-bp minisatellite probe (5’-GAGGGTGGCGGTTCT-3´) from the M13 bacteriophage and a simple DNA repeat sequence (GACA)_4_^5^. These markers have been widely used to discriminate between related strains of a wide variety of pathogenic microorganisms ^5^^,^^33^^-^^35^. As a negative control, we used a PCR mix with no DNA.

The PCR reaction mix of 50 pi included: 5 μl of 10X PCR buffer with 15 mM MgCI2, 3.0 μl dNTP mix (10 μmol/L each), 30 ng of primer, and 2.5 U of Taq DNA polymerase ^5^. Initial denaturation was at 94 °C for 20 sec followed by 35 cycles of denaturation at 94 °C for 20 sec, annealing at 50 °C for 1 min, amplification at 72 °C for 20 sec, and a final extension at 72 °C for 4 min in an MJ Research thermocycler. The PCR patterns were resolved by electrophoresis on 1.4 % agarose gels in Tris-borate-EDTA (TBE) buffer at 70 V for one hour and a half using the DNA 1 Kb plus DNA ladder as size reference (Fermentas). These were detected by ethidium bromide staining and were visualized on a BioDoc Analyze transilluminator (Biometra). The images were digitally recorded for further analysis.

### Fingerprint analysis

The PCR patterns obtained by the amplification of DNA fragments of different sizes for each isolate were termed as M13 and (GACA)_4_ genotypes. These fingerprints were analyzed by GelCompar II (Applied Maths NV, Kortrijk, Belgium) using the Pearson correlation coefficient, which considers each band pattern as a densitometric curve. Two dendrograms were built with the results of M13 and (GACA)_4_ using the UPGMA algorithm. These dendrograms and pairwise matrices calculated using Euclidean distance were then exported for posterior analysis.

Genotypes were clustered by their degree of similarity in each dendrogram. The isolates with identical band patterns were established as the same genotype and assigned the same number; those differing in one or two bands were considered closely related genotypes (with the same number but differentiated by a lowercase letter), and differing in three or more bands, as different genotypes (with different numbers). The identity of most of the strains (e.g. FOSC or FSSC), was determined in a previous work by TEF1-α partial sequence ^23^. Dendrogram annotation was done using the clustering classification, the isolation tissue, and the molecular identification at the species level.

### Hunter Gaston discriminatory index (HGDI) calculation

Hunter Gaston discriminatory index (HGDI) was calculated as proposed by Hunter, *et al*. and Sola, *et al*. ^36^^-^^37^:




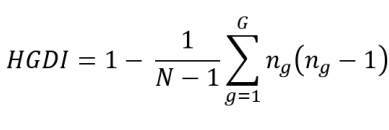




where N is the total number of strains in the population, is the total number of distinct patterns given by each genotyping method, and is the number of strains corresponding to the pattern . We took the clustering classifications obtained by M13 and (GACA)_4_ markers at the different levels of resolution given by the clusters (low resolution) and the genotypes (high resolution). For the sequencing methods (rDNA 28S and TEF1-α), we calculated the indexes grouping by the different haplotypes obtained using the Arlequín 3.5 software ^38^ as the haplotypic diversity. For the PCR-based identification, we grouped the results from the two pairs of primers 0X31/ 0X32 and Fusofor/Fusorev considering that they only provide a binary classification (i.e., presence/absence of amplification).

### Antifungal susceptibility tests

We evaluated a group of 44 isolates for their susceptibility to the in vitro activity of amphotericin b, itraconazole, and voriconazole using the disk diffusion test according to the CLSI M38-A methods as previously reported ^25^. Isolates were grown on PDA agar for a week at room temperature. Mycelia were resuspended with distilled water Into a 10 ml tube and sedimented for 20 min. The supernatant was collected and normalized to a concentration of 106 UFC/ml; 200 pi of this inoculum was added to RPMI agar plates with MOPS buffer (AES Laboratories, Paris, France) and dried for 15- 30 minutes. We performed an E-test following the manufacturer’s instructions (Etest®-AB Biomérieux). We placed on the agar plate two strips of antifungal with a concentration gradient in the range of 256 to 0.016 pg/ml opposite from the inoculum and incubated it at 28 ^º^C for 48 hours. Susceptibility values were recorded as the lowest antifungal concentration inhibiting the fungal growth.

### Clustering enrichment analysis

To test the clustering classifications obtained with the M13 and (GACA)_4_ markers and their associations with epidemiological variables we used a hypergeometric test. We evaluated the isolation tissue, the sequencing-based identification at the species level, the antimycotic sensibility in µg/ml, and the antimycotic phenotype (resistant, sensible-dose-dependent, and sensible) as reported in a previous characterization of these isolates ^25^. We controlled for false discovery rate adjusting by the Benjamini-Hochberg procedure ^39^. We also plotted some of these variable distributions using ggplot2 ^40^.

### Analysis of molecular variance (AMOVA) and correlation reference classification

The distance matrix obtained with the M13 marker was used in an AMOVA ^41^ analysis in search of population structures given the clustering and then compared to the TEF1-α and rDNA 28S haplotype reference classification. The correlation between the distance matrices computed with these methods was compared using a Mantel test ^42^.

The R code used to perform all the statistical analyses and cluster association figures can be found at https://github.com/welasqz/Fusarium_Genotyping

## Results

### *Fingerprinting clusters from M13 and (GACA)*_*4*_ *showed differences in their resolution.*

**Figure 1:**
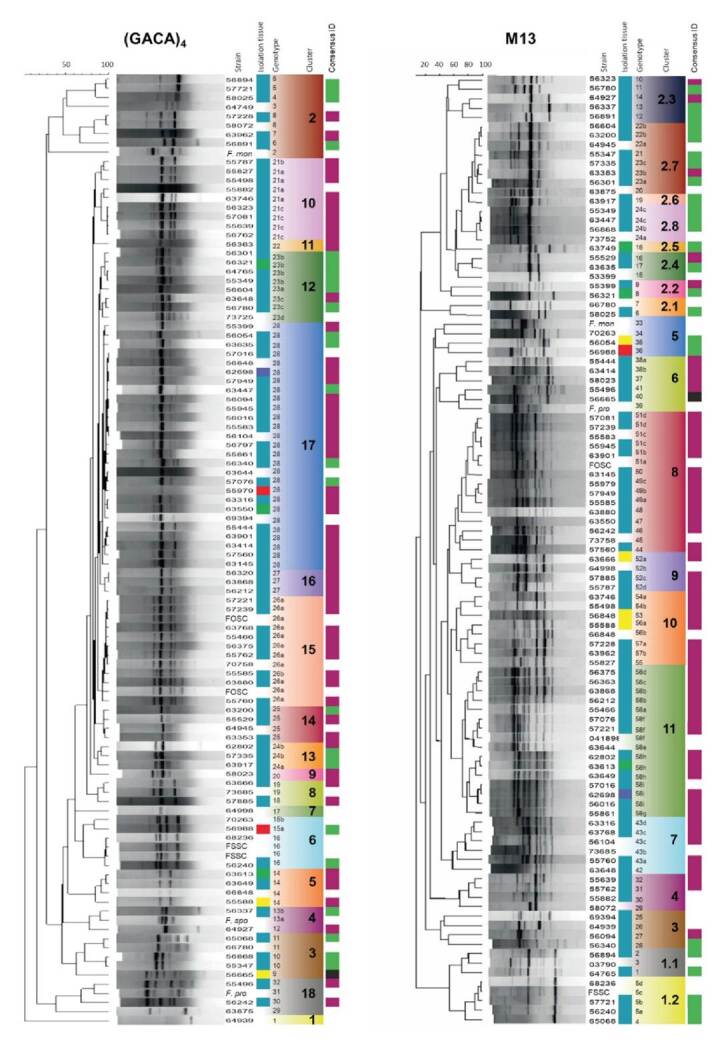
(GACA)4 and M13 dendrograms obtained by the UPGMA method using the Pearson correlation. The first column of each dendrogram correspond to the strain number. Several refence strains were included and abbreviated as FSSC (*F.solani* (Mart.) Sacc.), FOSC (*F. oxysporum* Smith & Swingle), *F.mon* (F. *moniliforme* J. Sheld. 1904), *F.pro* (F. *proliferatum* (Matsush.) Nirenberg 1976), and *F.spo* (*F. sporotrichioides* Sherb. 1915). The second column refers to the isolation tissue (blue: toenails, green: fingernails, purple: abdomen, red: cornea, and yellow: skin). The third column shows the genotypes classification with their number and clusters (differentiated by color and number). The last column refers to the consensussequencing identification (green: FSSC, purple: FOSC, and black: *Fusarium incarnatum*).

A total of 32 and 58 genotypes were revealed by the markers (GACA)_4_ and M13, respectively (figure 1).The M13 marker showed more power of resolution, and its band patterns were more complex and depicted a higher number of bands. The most frequent genotype by M13 was number 58, with 16 isolates, all belonging to FOSC. The second most frequent genotype was 51, with 6 isolates (of FOSC) followed by genotype 43 with 5 isolates (of FOSC), and genotypes 5 and 24 of FSSC and 52 of FOSC, with 4 isolates each one. Genotypes 22, 23, and 49 had 3 isolates each and the genotypes 38, 54, 56, and 57 had 2 isolates each. Unique genotypes (with only one isolate associated) were obtained for 48 (82.7%) isolates.

The most frequent genotype revealed by the (GACA)_4_ marker was number 28 assigned to 27 isolates followed by genotype 26 with 12 isolates; genotype 21 with 9; 23 with 8,14,16, and 25, each with 4; 5, 24, and 27 with 3, and genotypes 8,10,11,13,15, and 19 with 2 isolates each. Unique genotypes by (GACA)_4_ were obtained for 16 isolates (50%). Compared to M13, the majority of the (GACA)_4_ clusters had a mixture of different *Fusarium* species.

### *M13 and (GACA)*_*4*_ *showed the highest discriminatory power as compared to other genotyping methods for Fusarium*

HGDI values were computed for the different resolution levels obtained from the M13 and (GACA)_4_ markers, the sequencing-based genotyping using rDNA 28S and TEF1-α, and the PCR-based methods. Reference values ^37^ indicate a method is highly discriminatory if h>0.6, moderately discriminatory if 0.3<h<0.6, and poorly discriminatory if h<0.3. The highest discriminatory power was obtained by the genotype and high-resolution cluster level of M13 with 0.9970 and 0.9269, respectively. The (GACA)_4_ genotype and cluster classifications followed with 0.9156 and 0.8932, and then, M13 low-resolution clusters with 0.8693. TEF1-α had a value of 0.8305 followed by rDNA 28S with 0.7778, and, finally, the lowest value reported by PCR-based methods with 0.5235.

### The M13 marker was associated with epidemiological variables.

**Figure 2:**
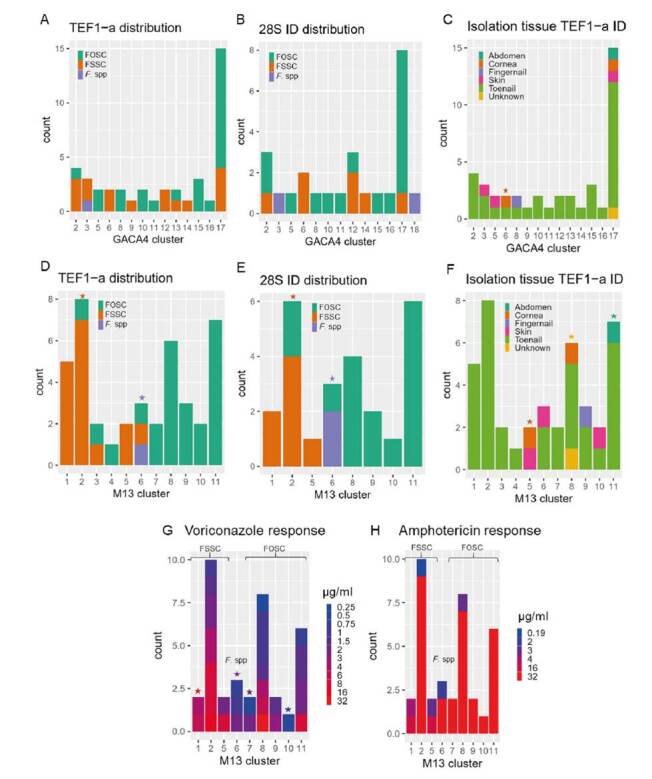
(GACA)4 and M13 association with several epidemiological variables. Cluster associations with the sequencing-based identification of the isolates for (GACA)_4_ (A, B) and M13 (D, E). Associations with the isolation tissue (C, F) for each marker clusters and associations between M13 and the response to antifungal compounds (G, H). M13 clusters 1 to 5 are enriched with isolates classified as FSSC, cluster 6 with *Fusarium* spp, and clusters 7 to 11 with FOSC. Significant hypergeometric values (p-adjusted <0.001) reported for category-mixed clusters indicating the significant category with the same color as in the histogram.

We plotted the clustering classifications computed with the M13 and (GACA)_4_ markers associated with several epidemiological variables as shown in figure 2. We looked for statistical associations among the levels of these variables and each cluster using a hypergeometric test. The M13 marker showed stronger associations with all the variables: 1) with the molecular identification given by TEF1-α and rDNA 28S, with clusters 1-5 linked with FSSC, with cluster 6 as a transition group (FSSC, FOSC, and *Fusarium* spp), and with clusters 7-11 containing strains identified as FOSC; 2) with isolation tissues, especially cluster 5 which grouped strains from the cornea and skin tissues, and cluster 11 containing all abdomen isolates; 3) with the voriconazole response showing resistant phenotypes associated with clusters 1-5 (FSSC) and susceptible-dose-dependent phenotypes in the 6-11 clusters (FOSC). The M13 marker was then taken forward as a useful genotyping method for *Fusarium*.

### M13 measured population structure and had a high correlation with the reference Fusarium identification method.

After exploring the M13 marker clustering when characterizing *Fusarium* populations from clinical samples, we assessed the genetic variability of the marker and its correlation with the genotyping obtained from sequencing- based methods. Haplotypes from TEF1-α and rDNA 28S were calculated for the FSSC and FOSC populations and inputted with the M13 Euclidean distance matrix to an AMOVA test. The highest haplotype diversity was found for FSSC with values of 0.8097 for rDNA 28S and 0.8917 for TEF1-α. FOSC had values of 0.5385 and 0.5435, respectively. Concatenated haplotypes were used as input data for the AMOVA and we found a population structure using the M13 fingerprint data with a p-value of 0.0099 and a φ statistic of 0.2509.

To confirm the correlation between the two methods, we performed a Mantel test on the distance matrices and found a strong correlation between them (p-value=0.02). These analyses established M13 as a discriminative marker for *Fusarium* molecular characterization maintaining the correlation and structure of the reference molecular identification and a higher discriminatory power.

## Discussion

*Fusarium* fungi are opportunistic pathogens causative agents of human mycoses; in agriculture, they cause a decrease in crop yields and economic losses. They are commonly characterized as a group of saprophytes in many environments and as characteristic inhabitants of the soil ^43^. Studies have called attention to the increasing cases of onychomycosis by non-dermatophyte fungi like *Fusarium* around the world ^7^; hypotheses explaining such an increase include better identification of these organisms as onychomycosis primary agents and the recent *Fusarium* spp. detection as emerging pathogens in growing immunocompromised populations ^7^.

*Fusarium* has great genetic variability and is difficult to classify ^26^^,^^44^. One of the major problems is the lack of diagnostic morphological characters, which makes the correct species identification and taxonomic classification very complex. However, the available molecular tools can improve the resolution at species level identification ^26^^,^^44^. The phylogenetic species recognition of *Fusarium* clinical isolates has been proposed based on the partial sequences of the TEF1-α, RPB1, and RPB2 genes ^32^. This method, also called multilocus sequence typing (MLST), is currently recognized as the best strategy for genetic population studies and to identify *Fusarium* species complexes ^8^^,^^32^. Our HGDI calculations indicate that M13 has a similar discrimination power to the MLST method documented for Fusarium, with 0.9970 for M13 vs 0.991 for MSLT ^32^. Besides, when a huge geographic diversity is involved, a large number of isolates of *Fusarium* from different environments, hosts, and tissues are available for biology, clinical, and evolutionary studies. Therefore, a simple, fast, cheap, and effective methodology is always required to select the most representative isolates for further studies. Here we confirmed that the M13 marker meets these qualifications.

More than 35.000 strains isolated from various substrates around the world are accessioned in the *Fusarium* Research Center (FRC) and the USDA- ARS National Center for Agricultural Utilization Research (NCAUR) Culture Collection, which makes this genus the best-preserved fungal group ^45^. Using this rich strain resource, extensive molecular phylogenetic studies have been conducted resulting in data covering most agriculturally and/or medically important species complexes. Despite these advances, a significant amount of diversity has yet to be explored and some species complexes are quite poorly characterized phylogenetically ^45^.

Here we were able to find a huge diversity of *Fusarium* genotypes (58 out of 101 isolates) using the M13 marker and the genotyping was consistent with the identification by TEF1-α for most of the isolates (figure 1) with a significant correlation between the two markers. We found associations between the clusters and multiple epidemiological variables (figure 2) such as the isolation tissue and the response to antimycotics, which supports the population structure given by the marker using AMOVA and demonstrates the utility of the marker in assessing the genetic variability of the isolates at the population level.

The most heterogeneous and basal genotypes were those belonging to FSSC, in agreement with the haplotypic diversity measurements from sequencing-based methods and other work ^46^^,^^47^. Then we found that the M13 cluster 6 is a transition group with multiple species followed by 5 clusters comprising the FOSC isolates. Noteworthy, most of the isolates we studied were identified as FOSC (53/77; 69%) and only 31% (24/77) as FSSC, which contrasts with other reports from Tropical areas (even with another one from Colombia) where FSSC was found to be the main human pathogen group of *Fusarium*^12^ probably due to the fact that FOSC is a prevalent plant pathogen and some genotypes rapidly disseminate and adapt in our geography. Noteworthy, many of the FOSC isolates had identical or closely related genotypes (e.g., clusters 7-11 in figure 1), and lower haplotypic diversity.

Besides the species complex identification, we showed associations between the M13 classifications and several epidemiological variables including the isolation tissue and the antifungal susceptibility. The associations with the isolation tissues indicated that the M13 cluster 5 was related to more unique tissues, such as cornea and skin, and that the genotypes isolated from these were closer to each other than the rest. In contrast, the most abundant tissues, such as toenails, had a broad distribution and diversity. When evaluating the response to antifungals, only the voriconazole presented a wide range of susceptibilities to test the associations with the classifications. We found that the response to voriconazole was associated with the species and, therefore, with the M13 classification. These results suggest that further research to develop new antifungal molecules is required for this group of fungi.

The M13 probe was established as a marker in 1987 to detect hypervariable DNA minisatellite regions in humans and animals ^48^. It has been used for studying the genetic epidemiology of pathogenic fungi ^49^ and was the first reported for typing isolates responsible for fungal keratitis belonging to FOSC and FSSC ^50^. Undoubtedly, considering the number of clinical isolates we characterized in this study and the low cost of the PCR- based methodology, the M13 marker is recommended to detect *Fusarium* genetic variability. We propose its use to choose the most diverse and/or frequent clinical isolates that should be analyzed using the MLST scheme and different omics such as genome sequencing. A local-blast analysis involving the 15-bp M13 sequence and the four *Fusarium* cured-genomes reported in the *Fusarium* Comparative Genomics Platform (FCGP) could reveal the extent of the genome coverage of this marker.
